# The Histology and Pathology of Reproduction*Abstract of a papcr read before the Section of Obstetrics, Ninth International Congress, Washington, D. C., Sept., 1887.

**Published:** 1888-01

**Authors:** Henry O. Marcy

**Affiliations:** Boston, Mass.


### ART III.—THE HISTOLOGY AND PATHOLOGY OF REPRODUCTION.*

BY HENRY O. MARCY, A. M., M. D., L.L D.
*Boston, Mass.*

Four years ago Italy mourned the loss of one of her most illustrious sons, Count Ercolani, of Bologna, a man like Germany's great scientist Virchow, alike celebrated as a leader in the great political struggles for the elevation of his people and an enthusiastic devotee of science. Edu-

*Abstract of a paper read before the Section of Obstetrics, Ninth International Congress, Washington, D. C., Sept., 1887.

cated in the school of the great Antoni Alessandriani, whose learned diligence he early imitated, he, in the most lamentable poverty of means, contributed to the foundation of those monuments of marvellous industry, the Museum of Comparative Anatomy and Veterinary Pathological Anatomy of Bologna. He obtained the highest offices of Bologna's most famous university, being several times President of the Medical Faculty and twice Rector of the University. Aided by the Government, he was enabled to erect new buildings and furnish the School of Veterinary Medicine with modern appliances for successfully carrying on original investigations in the department of Comparative Anatomy and Physiology. Although widely known by his many original contributions to science, he is especially deserving of, and receives, world-wide reputation for his long-continued investigations of the placental development in Vertebrates.

More than ten years since there first came to my notice an unpretending volume in French, published in Africa, a translation by Dr. Andrèini, of a certain portion of Prof. Ercolani's investigations, which, as a prize essay, had received an award from the Academy of Sciences at Paris.

In Harvard Library I found, in the Transactions of the Academy at Bologna, the original papers, with illustrations, published from time to time, having been presented as contributions to the Academy. Convinced of the singular value, as well as the originality of his work, I collated and presented to the English reading public the first edition, which included all his anatomical researches up to date of the publication, the last chapters of which were written specially for this edition. In 1880 was published a second and enlarged edition, which included also Ercolani's researches upon the pathological conditions of placental development, and a careful, analytical resumé of the whole subject, written by himself while yet suffering from the dire malady which speedily thereafter terminated his life. During all this period, I have myself carefully studied the changes incident to the uterus in the reproductive state in woman, and, as far as opportunity has been afforded me, comparative studies also in animals.

I am thereby convinced more than ever of the correctness of Professor Ercolani's teaching, and hold in admiration the singular ability and skill by which he arrived at conclusions so far reaching, and the remarkable clearness with which he demonstrated nature's uniform law of the unity of anatomical type in a simple and fundamental plan of embryonic nutrition and development.

The present occasion offers only the opportunity to review very briefly some of the more important deductions to be made therefrom.

In our own profession the study of the reproductive processes has been limited chiefly to the human species, and nothing is more complex or confusing, veritably a labyrinthian riddle, than the fully developed human placenta. The uterine mucosa in both woman and the lower animals had been studied with some care by many investigators for generations. To Malphegi, of the fifteenth century, we owe the first reliable demonstrations upon the uterine mucous membrane in woman and some species of the lower animals. He described the openings of the utricular glands into the uterine cavity, and observed that these glands, at least in the cow, increased in size during pregnancy. When we consider the lack of optical instruments in his day his observations are worthy of special comment. Little was added to this anatomical knowledge by scientists until within the present generation. Prof. Ercolani, as the trained comparative anatomist, early devoted his attention to a careful microscopic differentiation of the mucous membrane, with its glandular structure, in all the various species which he was enabled to bring under his observation. This naturally led to the investigation of the more complicated question of their function during pregnancy, and thus, little by little, was brought into differential study placentation.

The placenta is ordinarily sub-divided into diffused, multiple and single. Perhaps the simplest form of the first is found in the mare. Over the whole maternal portion of the uterus in this animal there is developed a series of secreting glands of follicular character, and into these it is easy to trace the villi of the fœtal portion of the placenta. A fœtal villus is little more than a vascular loop covered with epithelium. The glandular follicle is equally simple in anatomical construction, and also lined with epithelium, the one a villus of secretion, the other of absorption. This is the simplest possible type of the double structure of the placenta.

The multiple placenta of the cow offers the simplest form of this kind of placenta common to ruminants. The glandular maternal organ is here modified, although it does not lose its elementary form of a simple follicle. The modification consists chiefly in the uterine follicles being placed parallel to the surface, and sometimes superimposed upon each other instead of being disseminated vertically over the whole internal surface of the uterus, as in the diffused placenta. In the dog and cat the follicles are extraordinarily elongated into tubular glands, as it were, which are closely packed against the fœtal villi.

In the human species all that relates to the form itself of a glandular follicle is completely lost, but the fundamental parts of a secreting organ, that is to say the walls and cells, in a word, the gland and its secretion are persistent. The lesson taught by comparative anatomy lies in the following of these changes in the more simple form of placentation, and observing that in each the nutrition of the fœtus is provided for upon this simple plan. Having once determined with accuracy these facts, we are then, for the first time, prepared to devote ourselves intelligently to the discussion of placental development in woman.

It is sufficient here to make the simple statement of that which may be conceded as an accepted fact, that, in woman, the mucous membrane is reduced to a simple layer of epithelial cells, and that in impregnation there is a proliferation and destruction of these cells over the entire surface of the uterus. This destructive process of the epithelium on the internal surface of the uterus is in all cases indispensable, because this is what facilitates the setting up of the neo-formative changes from which will result the maternal portion of the placenta. This denudation of the internal surface of the uterus teaches that the formation of the decidua and the placenta is due neither to a tumefaction, nor to a transformation of the anatomical elements pre-existent at the time of conception in the uterine mucous membrane.

The neo-formative process of the maternal portion of the placenta, decidua serotina, consists in the production of new vessels which are distinguished from the ordinary uterine vessels; first, the arterial as well as the venous vessels have only a simple endothelial wall; second, on the external surface of their wall is elaborated a layer, more or less thick, of special cells not separable from the wall of the vessel. These are the so-called decidual or placental cells.

That the fœtal portion of the placenta is itself due to a neo-formative process has not been and cannot be questioned. It is from the constant relation established between these two parts of neo-formation that the placenta is developed. The manner in which this relation is established gives rise to the different forms of placenta known in the mammalia.

The various opinions which have been entertained concerning the origin of the cells which enter so largely into the formation of the maternal portion of the placenta have been exceedingly vague and uncertain. Prof. Turner, although concisely affirming with Owen that without decidua there is no formation of placenta, does not touch the important question of the origin of the decidua.

Prof. Kölliker confines himself to the remark that the decidua is a transformation of the uterine mucosa and not a new membrane or the product of an exudation as was once believed. It may be noticed, however, that Prof. Kölliker does not attempt to show what are the elements in the mucous membrane that compose the decidua, nor by what means they are transformed, although such an investigation should be of the highest interest for him, since the glandular follicles of new formation, as demonstrated by Prof. Ercolani, and confirmed by Turner, were declared by him to be only tumefactions of the pre-existing uterine mucous membrane, formed during pregnancy and disappearing after delivery.

The elementary and typical form of the two parts constituting the placenta in the mammifera is not imaginary, but is demonstrated in its simplicity by observation. It may be easily recognized in the fœtal portion in the villi of the chorion in the simpler forms of diffused placenta and, at the beginning of development, in the human species. In the maternal portion, the simple elementary form is found to be developed and maintained through the whole period of gestation in the uterus of certain viviparous fishes.

The manner in which the relation between the two parts is established may be by simple proximity, contact, or by intimate cohesion. When the relation is that of simple nearness, the maternal portion of the placenta manifestly presents the form of a glandular organ and has its limitation by the repetition of secretory villi upon the inner surface of the uterus which, uniting with each other in various ways, give rise to the formation of crypts, or glandular follicles, single, or compound, into which enter the absorbent villi of the chorion.

When the relation is more intimate and an adherence takes place between the two parts before mentioned, as in the single placenta, the glandular character is concealed by the fact of the adhesion, but the fundamental condition remains constant. The contact in this case is direct, between the vessel of the absorbent villus and the epithelium of the secretory villus which is never lost. Only two very simple changes occur in the fundamental parts of the placenta when single, and they are the factors of the manifold differences which are observed ; first, the loss of the epithelium of the absorbent villus, which is not important since there is established direct contact of the vessel of the fœtal villus, with the secretory epithelium of the maternal villus and this fact is constant. Second, the dilatation, or ectasia of the vessel in the maternal villus, and this is remarkable only in the placenta of the quadrumanna and in woman.

The chorial villi away from the placental site in the atrophic state of advanced fœtal development may still be seen covered with epithelium although shrunken and atrophied. In the intimate cohesion with the maternal villi, in the developing placenta the epithelium covering the chorial villus is lost. This may be easily demonstrated by observations upon the placenta in abortions, where the death of the fœtus has for some time preceded the separation of the placenta. Here the fœtal villus is shrivelled and shrunken and is easily defined as distinct from the maternal villus with its layer of actively growing placental cells.

The formation of the lacunæ therefore precedes the formation of the tufts of the villi and cannot be an effect, since they are observed before the existence of the cause assigned by Kölliker. Still further and more conclusive evidence upon the formation of the lacunæ, independent of the presence of the fœtal villi, may be found in the structure of the so-called uterine decidua in cases of extra-uterine pregnancy, and there is formed the exact and isolated anatomical structure of the maternal portion of the placenta in which exist lucanæ through ectasia of the vessels, without any trace of the fœtal villi Kölliker himself teaches, that the short utero-placental arteries, when they penetrate into the placenta, lose their distinctive anatomical characteristics, that is, they no longer have muscular fibres, or elastic elements, and their whole wall is formed by an endothelial layer covered with a thin sheath of connective tissue which disappears and blends with the decidua serotina.

He states, also, that the veins are no longer to be distinguished from the arteries and all traces of both are lost in the interior of the placenta, where alone the large lacunæ are found.

In 1876, De Sinètz called attention to an important demonstration in its bearing upon the above veins. He noticed that the cells of the decidua in woman form a circular sheath about the placental vessels. However, if the facts observed by him in the completely developed human placenta, harmonize with those already noticed early in its development, when there is seen a minute network of capillaries, that become ectasic in the midst of the cells of the decidua or placental serotina, we shall be convinced that it is not the walls of the utero-placental vessels which are lost in these cells, as was indicated by Prof. Kölliker, but that so enormous a dilatation has taken place as to render it difficult to perceive the endothelium and to separate it from the placental cells, that are elaborated by their external walls.

The belief that the lucanæ are really large cavities, as they seem to be microscopically, and not the maternal vessels greatly dilated, is still generally taught and, through this belief, two other deceptive appearances are accepted as actual truths, that the chorial villi float in the maternal blood, and that the epithelium covering them appertains to the fœtus instead of the mother. Setting aside these fundamental errors, many facts already observed by able anatomists in the human placenta, which have remained doubtful, or were wrongly interpreted, now receive, under the teaching of Ercolani, a clear and precise explanation.

Briefly reviewed, these changes take place in woman in pregnancy. The mucous membrane, which consists of a simple layer of epithelium, closely attached to the sub-jacent muscular walls, disappears.

It is replaced by a layer of decidual cells, proliferated from the vascular network of the uterine wall. The utricular glands are not destroyed but, on the contrary, are increased in size. The constant secretion therefrom forms openings through the decidua vera, which may easily be traced by the unaided vision, as a sieve-like perforation through the proliferation of the serotinal cells at the placental site. These glands are, by the pressure from their own obstructed secretion, dilated and altered, and this forms the strata called by Kölliker the non-caducous portion of the placenta. Traces of these glands may be observed, even to the termination of pregnancy, but none whatever are found in the caducous stratum of the placenta, as defined by Kölliker, where it would be expected that they would be found, if as claimed by him, the placenta is a transformation of the uterine mucosa. All anatomists agree that the stratum, where the placenta is formed, is developed from a mass of cellular decidual elements, traversed by a vascular network with endothelial walls, only during the first months of pregnancy, and that among these elements the fœtal villi are buried. During the first three months of pregnancy the fœtal villi can be separated from the maternal portion without a laceration of the latter, in the same way as in the mammiferous non-deciduates.

The second period of development, by the universal consent of anatomists, begins with the establishing of two facts, the rapid and exuberant proliferation of branches from the trunks of the fœtal villi and the ectasic process in the network of the maternal vessels. These conditions taking place contemporaneously in a limited and circumscribed space, it must necessarily follow, as the result of simple physical and mechanical law, that the branches of the proliferating villi should press against the endothelial walls of the vessels, which are at the same time thinned and dilated, and the ultimate result is that the walls of the villus, at first bent in simply towards the internal cavity of the dilating vessel, must, as the process of aneurism becomes more and more pronounced, completely invest the villus, aided also by the tension exercised upon the walls of the vessel by the rapidly proliferating branches of the villi. Weber and Virchow observed this condition in abortions, describing the tufts of the villi as making a complete hernia in the maternal vessel. As a result thereof the epithelial covering of the chorial villus disappears.

Intimate union with the introflected wall of the maternal vessel lined with its layer of decidual cells ensues, and the picture of the villi swimming in the lacunæ is complete.

The belief that the villi in the placenta of woman floats in the blood of the lacunæ generated the physiological error that the nutrition of the fœtus, not only in the human species, but in all mammals, takes place through an asmatic exchange of the two bloods, although in the case of diffused placenta the facts openly contradict such an assertion.

Moreover, in all cases where the placenta is single, the vessel of the absorbent villus, the fœtal, never comes in contact either with the blood or with the wall of the maternal vessel.

There is always interposed between the walls of the two vessels, and consequently of the two bloods, a cellular layer which is the epithelium of the maternal villus, and that this is secretory is confirmed by the obvious glandular appearance which is observed in many animals in the maternal portion of the placenta when it has the diffused form.

Prof. Ercolani sums up with the following conclusions: In all the vertebrates the nutritive material which is to serve for the growth of the fœtus is furnished by the mother. In mammals it is supplied by the maternal portion of the placenta, gradually, as the fœtus developes. In the oviparous vertebrates, the material, in the quantity necessary for the development of the fœtus, is emitted in a mass from the mother in the egg. In the mammiferous, as in the oviparous animals, the absorbent or fœtal part does not change, and it is by means of an absorbent villus, more or less complicated, that the material elaborated by the mother is conveyed to the fœtus. There is, therefore, but one law, a physiological modality, that governs the nutrition of the fœtus in all the vertebrates.

